# Supplementary material for: Integration of in vitro and in silico approaches enables prediction of drug-induced liver injury
Source: Arch Toxicol. 2026 Feb 17;100(5):2029–46. doi: 10.1007/s00204-026-04305-2 (PMC13086711; doi:10.1007/s00204-026-04305-2)
Supplement: Supplementary file 1 — Supplementary Material 1 [file 204_2026_4305_MOESM1_ESM.docx]

# Supplementary Material

|  |  |  | |  | |  |  |  |  |
| --- | --- | --- | --- | --- | --- | --- | --- | --- | --- |
| **Supplementary Table 1: Predictive performance metrics of various DILI prediction strategies.** Calculations were based on distinguishing No- from Most-DILI concern and Clinical Development Failure drugs, and assuming that mifepristone and primidone are DILI-positive, despite being classified No-DILI concern in DILIrank, for the reasons outlined in text. Ideal thresholds are based on Youden’s index(YOUDEN 1950). | | | | | | | | | |
| **DILI Prediction**  **Strategy** | **Parameter** | | **Classification**  **Threshold Value** | | **Sensitivity**  (No vs Most/  Clinical Dev) | | **Specificity** (No vs Most/  Clinical Dev) | **Balanced Accuracy** | **ROC AUC** |
| 1: Screening  heuristics | LogP | | 3 | | 61.6% | | 39.1% | 50.4% | 55.8% |
|  | Dose | | 100 mg | | 79.5% | | 82.6% | 81.0% | 89.8% |
|  | Rule of Two | | LogP >=3, Dose >= 100 mg | | 45.5% | | 91.3% | 68.4% | - |
|  | *In vivo* Cmax | | 1.1 µM | | 82.1% | | 93.5% | 87.8% | 93.1% |
|  | *In silico* Cmax | | 1.1 µM | | 81.2% | | 91.3% | 86.3% | 90.5% |
|  | Lowest *In vitro* toxicity (incl. BSEP) | | 29.44 µM  (ideal) | | 70.5% | | 66.7% | 68.6% | 67.34% |
| 2a: *In vitro* hepatotoxicity  excl. BSEP  (retrospective) | Ratio of *in vivo* Cmax to functional *in vitro* toxicity (excl. BSEP) | | 0.25 (mechanistic) | | 50.9% | | 100% | 75.4% | 96.5% |
|  |  |  | 0.011  (ideal) | | 97.3% | | 84.2% | 90.8% |  |
| 2b: *In vitro* hepatotoxicity  incl. BSEP  (retrospective) | Ratio of *in vivo* Cmax to lowest *in vitro* toxicity (incl. BSEP) | | 0.25 (mechanistic) | | 55.4% | | 100% | 77.7% | 96.8% |
|  |  |  | 0.024  (ideal) | | 92.9% | | 89.1% | 91.0% |  |
| 3a: *In vitro* hepatotoxicity  excl. BSEP  (prospective) | Ratio of *in silico* Cmax to functional *in vitro* toxicity (excl. BSEP) | | 0.25 (mechanistic) | | 50.0% | | 94.7% | 72.4% | 92.3% |
|  |  |  | 0.017  (ideal) | | 91.1% | | 84.2% | 87.6% |  |
| 3b: *In vitro* hepatotoxicity  incl. BSEP  (prospective) | Ratio of *in silico* Cmax to lowest *in vitro* toxicity (incl. BSEP) | | 0.25 (mechanistic) | | 55.4% | | 95.7% | 75.5% | 93.7% |
|  |  |  | 0.017  (ideal) | | 92.0% | | 81.2% | 86.6% |  |
| 4: BSEP inhibition alone | Ratio of *in vivo* Cmax to BSEP IC50 | | 0.25 (mechanistic) | | 42.0% | | 100% | 71.0% | 96.4% |
|  |  |  | 0.012  (ideal) | | 92.6% | | 92.0% | 92.3% |  |
| 5: Bile acid model simulations | Duration of Bile Acid Elevation above two-fold | | 100 min | | 22.2% | | 100% | 61.1% | 61.7% |

| **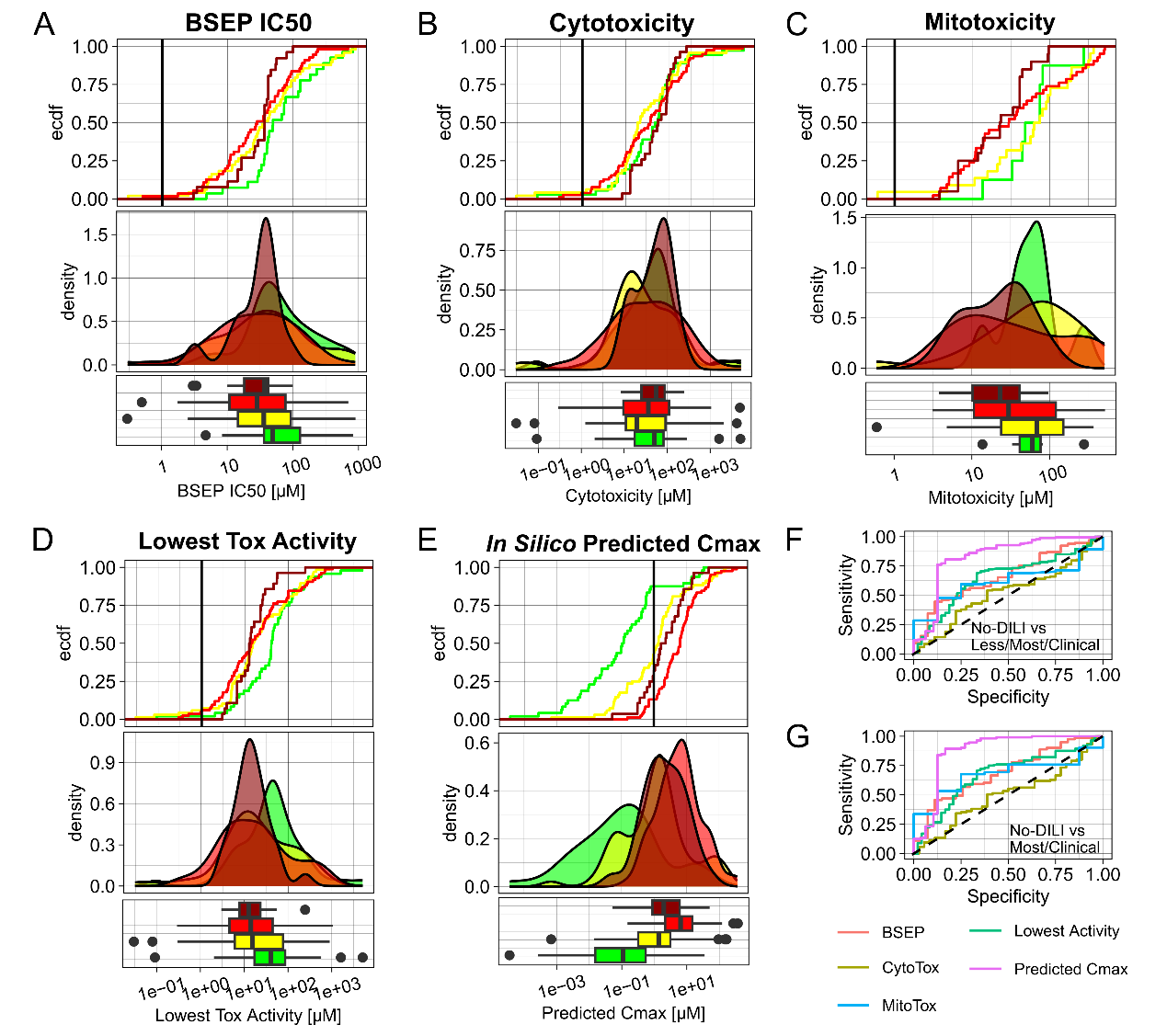** |
| --- |
| **SI-Fig. 1: DILI predictivity of i*n vitro* toxicity and *in silico*-predicted Cmax values alone.** Cumulative distribution, probability density and boxplots of different properties of drugs of the different DILIrank classes: *In vitro* BSEP IC50 (A), cytotoxicity (B), mitochondrial toxicity (C), the lowest toxicity potency per compound (D) and *in silico*-predicted Cmax values (E). ROC curves of the various parameters for distinguishing No-DILI compounds from Less-/Most-DILI/Clinical Development Failures (F) or only from Most-DILI/Clinical Development Failures. (G). |

| **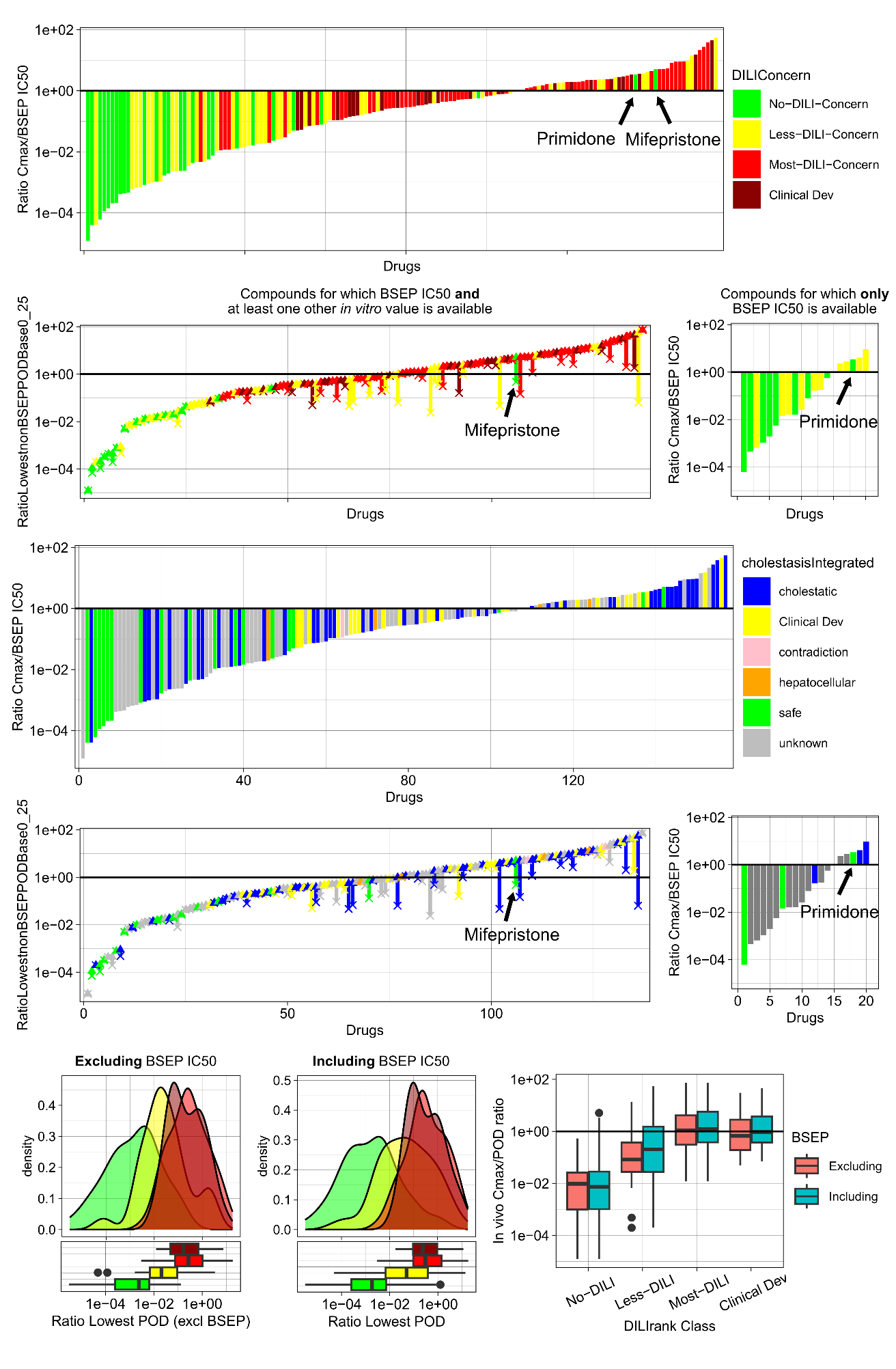** |
| --- |
| **SI-Fig. 2:** **DILI patterns of drugs with BSEP inhibition data.** Ratio of *in vivo* Cmax to BSEP IC50 values of each drug (A). Changes in Cmax to toxicity ratios when including BSEP IC50 values for compounds that have at least one functional *in vitro* toxicity values (B). Ratio of Cmax to BSEP IC50 values of compounds that have no functional *in vitro* toxicity value (C). |

| 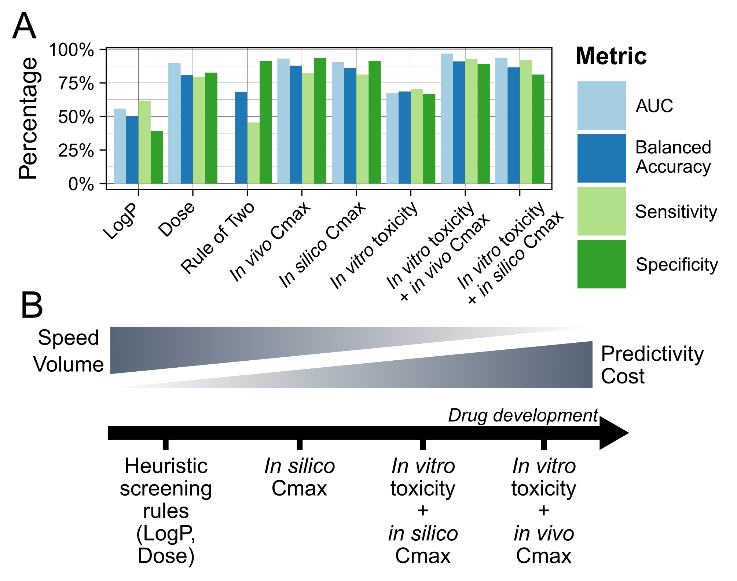 |
| --- |
| **SI-Fig. 3: Summary of predictivity evaluations of various DILI prediction strategies.** *In vitro* activity refers to the lowest *in vitro* measured toxicity per compound, including cytotoxicity, mitochondrial toxicity and BSEP inhibition (A). Ideal thresholds based on Youden’s index are used. Rule of Two does not possess a ROC AUC value, due to absence of drug risk ranking. Schematic representation of the role of different DILI prediction strategies in the drug development timeline (B). |

| **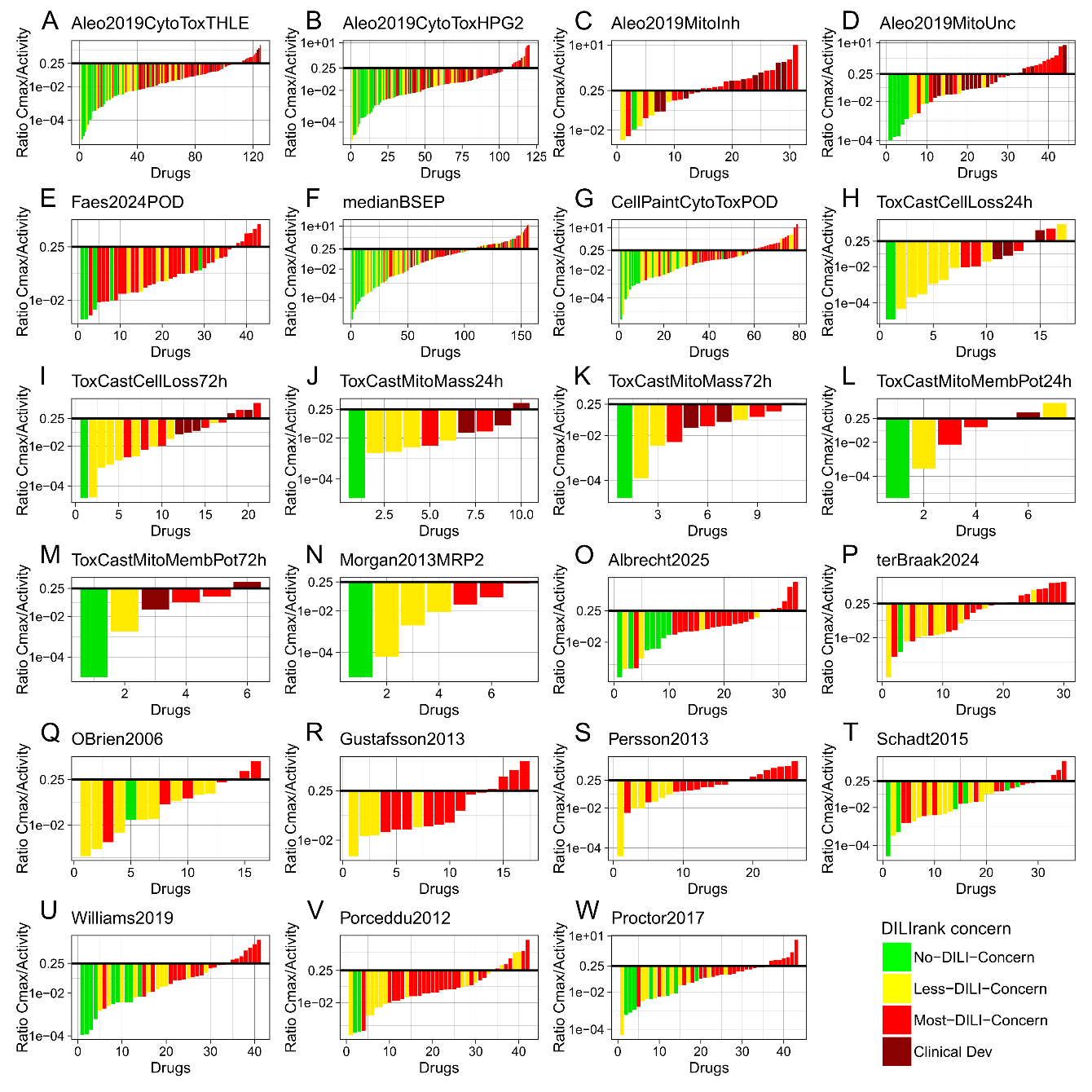** |
| --- |
| **SI-Fig. 4: Individual results of each dataset integrated in this study.** Ratio of *in vivo* Cmax to lowest functional *in vitro* toxicity of each drug for each *in vitro* toxicity dataset integrated in this study (A-W). |
